# Factors associated with higher hemoglobin A1c and type 2 diabetes-related costs: Secondary data analysis of adults 18 to 64 in Texas with commercial insurance

**DOI:** 10.1371/journal.pone.0289491

**Published:** 2023-09-08

**Authors:** Marcia G. Ory, Gang Han, Sagar N. Jani, Lixian Zhong, Elena Andreyeva, Keri Carpenter, Samuel D. Towne, Veronica Averhart Preston, Matthew Lee Smith

**Affiliations:** 1 Center for Community Health and Aging, Texas A&M University, College Station, Texas, United States of America; 2 Department of Environmental and Occupational Health, School of Public Health, Texas A&M University, College Station, Texas, United States of America; 3 Department of Epidemiology and Biostatistics, School of Public Health, Texas A&M University, College Station, Texas, United States of America; 4 College of Pharmacy, Texas A&M University, College Station, Texas, United States of America; 5 Department of Health Policy and Management, School of Public Health, Texas A&M University, College Station, Texas, United States of America; 6 School of Global Health Management and Informatics, University of Central Florida, Orlando, Florida, United States of America; 7 Disability, Aging, and Technology Cluster, University of Central Florida, Orlando, Florida, United States of America; 8 Southwest Rural Health Research Center, Texas A&M University, College Station, Texas, United States of America; 9 Blue Cross Blue Shield of Texas, a subsidiary of Health Care Service Corporation, Richardson, Texas, United States of America; National Healthcare Group, SINGAPORE

## Abstract

**Objective:**

This study will identify factors associated with higher hemoglobin A1c (A1c) values and diabetes-related costs among commercially insured adults in Texas diagnosed with type 2 diabetes.

**Research design and methods:**

This secondary data analysis was based on claims data from commercially insured individuals 18–64 years of age residing in Texas with diagnosed type 2 diabetes during the 2018–2019 study period. The final analysis sample after all the exclusions consisted of 34,992 individuals. Measures included hemoglobin A1c, diabetes-related costs, Charlson Comorbidity Index, diabetes-related complications, rurality and other socioeconomic characteristics. Longitudinal A1c measurements were modeled using age, sex, rurality, comorbidity, and diabetes-related complications in generalized linear longitudinal regression models adjusting the observation time, which was one of the 8 quarters in 2018 and 2019. The diabetes-related costs were similarly modeled in both univariable and multivariable generalized linear longitudinal regression models adjusting the observation time by calendar quarters and covariates.

**Results:**

The median A1c value was 7, and the median quarterly diabetes-related cost was $120. A positive statistically significant relationship (p = < .0001) was found between A1c levels and diabetes-related costs, although this trend slowed down as A1c levels exceeded 8.0%. Higher A1c values were associated with being male, having diabetes-related complications, and living in rural areas. Higher costs were associated with higher A1c values, older age, and higher Charlson Comorbidity Index scores.

**Conclusion:**

The study adds updated analyses of the interrelationships among demographic and geographic factors, clinical indicators, and health-related costs, reinforcing the role of higher A1c values and complications as diabetes-related cost drivers.

## Introduction

Type 2 diabetes is a costly and potentially preventable illness affecting millions throughout the US [1]. The total annual cost associated with diabetes was most recently estimated in 2017 to be approximately $327 billion in direct medical costs as well as another $90 billion in lost productivity [1]. Nationally, a more than 2-fold increase in annual medical costs was incurred by people with diagnosed diabetes [1]. Unmanaged blood glucose levels can cause short- and long-term complications associated with diabetes [2]. Such complications include, but are not limited to, diabetic ketoacidosis, chronic kidney disease including nephropathy, retinopathy, neuropathy, foot ulcers, and skin ulcers [3].

Given the magnitude of the estimated cost burden nationwide along with anticipated increases since the last national estimates, there is a critical need for continued study of diabetes-related costs and associated factors. Moreover, identifying those factors influencing possible variation in A1c that may be associated with variation in diabetes-related costs would be of value in planning clinical interventions. For example, some past work has suggested that lower socio-economic status (SES) may be associated with higher A1c [4, 5]. As is common in medical claims data, detailed information on income may not be collected, meaning the use of proxies for SES, even aggregate measures based on location, may be warranted. For example, geographic location is a key factor for study [6], as higher rates of poverty and lower income [7] were identified in rural areas of the US. Rural areas are of particular interest, given the increased prevalence of diabetes risk factors (e.g., obesity, smoking, high blood pressure) combined with the barriers to healthcare services and transportation [8, 9]. Additionally, we need to know more about how health insurance coverage, which dictates services and care received, impacts health care utilization and costs [9]. Additionally, demographic and clinical factors have been associated with costs, such as age [10], gender [10], comorbidities [11], diabetes complications [11], and A1c levels [12].

The American Diabetes Association (ADA) recommends that the target A1c for most non-pregnant US adults with type 2 diabetes should be <7%, signifying controlled blood glucose levels for the individual [13]. Prior evidence suggests that even small reductions in A1c can reduce the diabetes-related healthcare costs (i.e., a 1% reduction has been shown to reduce costs by 6.9% [12]. Therefore, understanding factors related to A1c levels is important for helping to manage diabetes complications and related costs.

### Approach and objective

While analyses at the national level add critical insight, policymakers and key stakeholders can use state-specific data [14] to inform policy-relevant planning and forecasting at more local levels. Such information is valuable in estimating future expenditures and responding to anticipated needs. Further state-based analyses can be especially relevant in identifying high-priority subgroups that may be more at-risk within a given state. For example, states with a relatively larger share of rural residing individuals may benefit from analyses that highlight potential variation in costs by rurality. Such studies that tailor detailed cost-related analyses more closely aligned with state priorities, may provide highly relevant and actionable data that can guide multi-sector planning throughout a given state and be updated to reflect changing priorities over time.

As a specific example, Texas, home to a diverse population residing in a wide range of geographic areas (e.g., remote rural areas, border cities, large metropolitan areas), provides a unique setting for the current study. Diagnosed diabetes costs an estimated $26 billion annually in direct medical expenses and other indirect costs [15]. Given this large diabetes burden, efforts are needed to better understand the drivers of unmanaged diabetes and diabetes-related costs among population subsets. Thus, we sought to investigate potential variation in medical costs associated with diagnosed type 2 diabetes across clinical-related characteristics (e.g., A1c, comorbidities), sociodemographic characteristics (e.g., sex, age), and geographic location of a person’s residence (e.g., rurality) using a Texas-wide commercial insurance dataset. The specific objectives of this study were to: (a) describe the characteristics of commercially insured among adults diagnosed with type 2 diabetes between the ages of 18 and 64 residing in Texas; (b) identify factors associated with their higher A1c values; and (c) identify factors, including A1c values, associated with higher diabetes-related costs. This study adds updated analyses of both clinical indicators such as A1c, cost-related factors, and associated cost estimates for a large southern state.

## Methods

### Data source and sample selection

**Fig 1:**
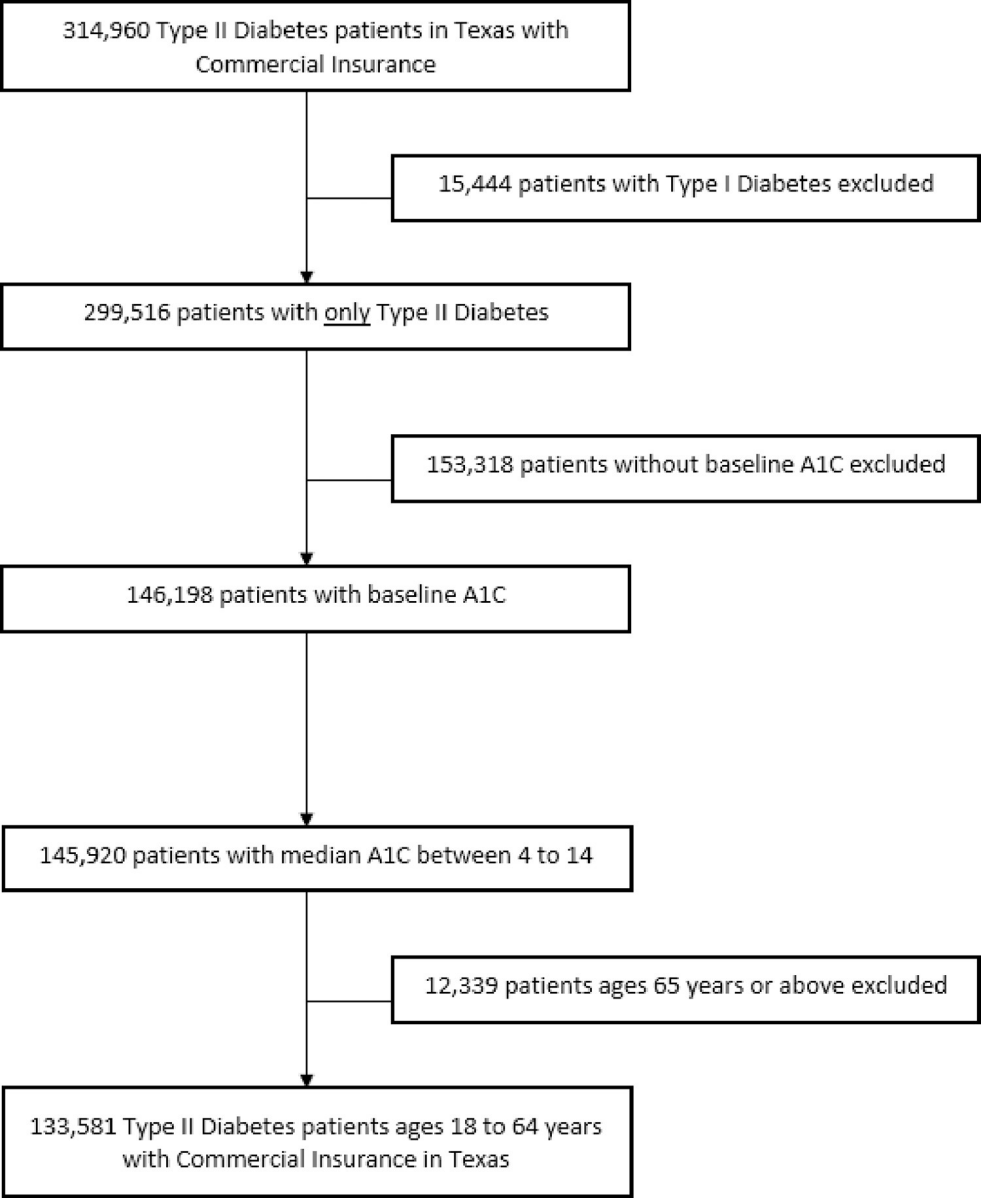
Consort diagram of patient flow.

We used 2018–2019 commercial claims data provided to us by a commercial insurer with a significant presence as the largest private health insurer in Texas. We restricted our analysis to commercially insured individuals 18–64 years of age residing in Texas with an ICD type 2 diabetes diagnostic code during the study period. We restricted the analysis to individuals with valid A1c values between 4 and 14 reported during the study period and provide a supplemental table to show that this restriction eliminated less than 1% of reported A1c values. To identify individuals with type 2 diabetes we used the *International Classification of Diseases*, Tenth Revision (ICD10), code E11 [16]. Fig 1 illustrates the final analysis sample after all the exclusions resulting in 133,581 individuals with type 2 diabetes.

### Theoretical framework

Our study goals are influenced by the desire to document and ameliorate health disparities in chronic disease care. The National Institute on Aging Health Disparities Framework (https://www.nia.nih.gov/research/osp/framework) designates priority populations and illustrates different analytical levels associated with illuminating disparities research: environmental, sociocultural, behavioral, and biological. Our research explicitly seeks to understand health care in rural populations and focuses on the interplay of environmental, social and biological factors. While we recognize the importance of behavioral factors in health and health care, given the current data set we were not able to include in-depth health behaviors in our model. However, the available variables fit nicely into the Anderson model [17] with sex as Predisposing, rurality as Enabling, and age, comorbidity, and diabetes complications as Need factors.

### Measures

#### Hemoglobin A1c

Diabetes control over time is often measured by the glycated hemoglobin (A1c) test, which measures the average plasma glucose in the previous eight to twelve weeks [18]. Per study inclusion criteria recommended by clinical colleagues, A1c values ranged between 4 and 14 to ensure feasible scores were included and account for variability in the sensitivity of measurement instruments across clinical settings. For all descriptive statistics, to combine values across the eight calendar quarters, the median A1c values were used for patients with two or more A1c values over the study period. In longitudinal multivariate models, the mean A1c value per quarter (if available) was treated continuously.

#### Diabetes-related cost

Rather than using the total of all medical related cost, we chose to specify only those diabetes-related costs, given our aim in assessing diabetes-specific costs among those with type 2 diabetes. Costs attributed to diabetes care were obtained from administrative claims records per patient based on ICD-10 code E11 [16] in primary, secondary, or tertiary diagnosis. Total diabetes-related costs were based on multiple health care utilization cost categories including inpatient stay, outpatient visits, generalist visits, and specialist visits. Emergency Department (ED) visits were also reported, including ED outpatient visits (a subset of outpatient visits) and inpatient ED admissions (a subset of inpatient visits). Medication costs were not included in our data set and hence not part of this analysis. For the descriptive analysis, the costs were aggregated at the patient-year level. Costs were adjusted by total annual days of eligibility to calculate costs per patient per quarter, which was used in longitudinal multivariate models. All costs were inflated to 2019 US dollars using the US Bureau of Labor Statistics Consumer Price Index for medical care [19].

#### Charlson Comorbidity Index (CCI)

Patient comorbidities were provided for patients based on their International Classification of Diseases Version 10 (ICD10) diagnosis codes, with scores ranging from 0 (no comorbidities) to 17 for each quarter [20]. The maximum CCI value per patient over the 8-quarter study period was used for descriptive statistics. In longitudinal multivariate models, the mean CCI value per quarter (if available) was treated continuously.

#### Diabetes-related complications

Short- and long-term diabetes-related complications were documented in the administrative claims data. Examples of short-term complications included hyperglycemia, hypoglycemia with coma, hyperosmolarity with coma, and hyperosmolarity without nonketotic hyperglycemic-hyperosmolar coma [3]. Examples of long-term complications included chronic kidney disease including nephropathy, retinopathy, neuropathy, foot ulcers, and skin ulcers [3]. The presence of any short- and/or long-term over the 8-quarter study period were scored as 0 (no complication) or 1 (1+ complication), respectively. Then, these two binary variables were combined to create a 4-category variable scored 0 (no complications), 1 (only short-term complications), 2 (only long-term compilations), and 3 (both short- and long-term complications).

#### Rurality

Rurality was measured based on the patient’s county of residence at baseline, which was linked to a database maintained by the National Center for Health Statistics (NCHS). The database used was the NCHS Urban-Rural Classification Scheme for Counties [21]. NCHS Urban-Rural Classification Scheme includes 6 levels across metropolitan areas (large central metro, large fringe metro, medium metro, small metro) and non-metropolitan areas (micropolitan, non-core) [21]. For all analyses, these 6 categories were collapsed to create a binary variable that enabled distinguishing between metropolitan counties (large central metro, large fringe metro, medium metro, small metro) and non-metropolitan counties (micropolitan, non-core).

#### Sociodemographic characteristics

Patients’ baseline age and sex were used in analyses. Age was a continuous variable ranging from 18 to 64 years. Sex was treated categorically. As indicated in the limitations, race/ethnicity was not systematically recorded in the data set and hence could not be included in the analyses.

### Statistical analyses

The raw data were in a longitudinal format per quarter. To generate individual-level summary statistics, we calculated summary statistics from the 8 quarters in 2018 and 2019 for each participant, including the baseline and median A1Cvalues, median age, highest CCI over the two years, short- and long-term diabetes-related complications over the two years, rurality, and sex. Descriptive statistics including median with interquartile range for continuous variables, and frequency with percentage for discrete variables were provided for all participants. Variables were compared by complication type (i.e., no complications, short-term complications only, long-term complications only, and both short and long-term complications) and rurality (i.e., metro, non-metro). Kruskal-Wallis test and Pearson’s Chi-square test were used for testing continuous and categorical variables respectively.

Distributions of the two outcome variables (i.e., A1c and type 2 diabetes-related cost) were found to be highly skewed to the right. Different transformations were attempted, including log, square root, and box cox transformations. We used the log transformation because it is sufficiently operationalizable, and in the plot of A1c and cost, both variables showed bell-shaped distributions. The raw A1c was between 4 and 14, and the raw cost value was greater or equal to 0. To regulate the data on the log scale, we set the log transformation such that: (1) if the cost was 0 in the raw scale, the cost was also 0 on the log scale (i.e., log of cost = log(cost+1)), and (2) if A1c was 4 in the raw scale, the A1c was 0 on the log scale (i.e., log of A1c = log(A1c-3)).

Longitudinal A1c measurements were modeled using age, sex, rurality, CCI, and diabetes-related complications in generalized linear longitudinal regression models adjusting the observation time, which was one of the 8 quarters in 2018 and 2019. We accounted for the cluster effect of observations of the same individual using generalized estimating equations for longitudinal models [22]. Developed by Liang and colleagues [23], a generalized estimating equation can estimate the parameters of a generalized linear model with possible correlations between observations from different timepoints in longitudinal data. The type 2 diabetes-related costs were modeled in both univariable and multivariable generalized linear longitudinal regression models adjusting the observation time by calendar quarters and covariates. Among the covariates, A1c, CCI, and complications are time-dependent, while other covariates including age, sex, and rurality are not significantly time-dependent. Rurality and diabetes-related complications were also tested in univariable models of type 2 diabetes-related cost. To account for a possible non-linear relationship between A1c and cost, we tested polynomial regression terms of A1c, including the linear and quadratic terms, as well as the interaction between A1c and time. A P-value ≤ of 0.05 was considered statistically significant. All analyses were conducted using SAS software, version 9.4 (SAS Institute, Cary, NC).

### Human subjects

This study consists of a secondary data analysis of data from health claims records. It was ruled as “non-human subjects” research by the Texas A&M Institutional Review Board since the data was deidentified. However, we recognized that the primary data set did involve humans.

## Results

Table 1 contains descriptive statistics for our analysis sample, including mean, median, interquartile range, sample size, frequency, and percentage. The mean age was approximately 52 years, 56% were men, and 12% resided in non-metro areas. The median CCI score for participants was 1, although 35% had scores of 2 or higher. The median A1c value was 7, and the median quarterly type 2 diabetes -related cost was $120. Compared to having no diabetes-related complications, having diabetes-related complications was correlated with older age, higher CCI scores, higher A1c values, and higher diabetes-related costs. Compared to non-metro areas, metro area residents had 2 to 3 percent more short-term and long-term complications.

**Table 1:** Sample characteristics with p-values by diabetes related-complication type and rurality*.

Variable	Level	All with Complication	Complication No	Complication Short only	Complication Long only	Complication Short & Long	Complication P-value	All with Metro Status	Metro	Non-Metro	Metro P-value
**Age**		51.82; 54, (46, 59) N = 128791	51.85; 54, (46, 59) N = 59898	50.66; 52, (45, 58) N = 34471	53.39; 55, (49, 60) N = 19749	52.29; 54, (47, 59) N = 14673	< .0001	51.70; 54, (46, 59) N = 133581	51.66; 54, (46, 59) N = 117680	52.01; 54, (47, 59) N = 15901	< .0001
**Sex**	Female	56691 (44%)	26958 (45%)	15131 (44%)	8183 (41%)	6419 (44%)	< .0001	58747 (44%)	51870 (44%)	6877 (43%)	0.11
	Male	72090 (56%)	32936 (55%)	19337 (56%)	11564 (59%)	8253 (56%)		74824 (56%)	65802 (56%)	9022 (57%)	
	Other	10 (0%)	4 (0%)	3 (0%)	2 (0%)	1 (0%)		10 (0%)	8 (0%)	2 (0%)	
**Charles Index (continuous)**		1.76; 1, (1, 2) N = 124239	1.38; 1, (1, 1) N = 55346	1.46; 1, (1, 2) N = 34471	2.46; 2, (1, 3) N = 19749	2.96; 3, (2, 3) N = 14673	< .0001	1.76; 1, (1, 2) N = 124404	1.77; 1, (1, 2) N = 109769	1.71; 1, (1, 2) N = 14635	< .0001
**A1c median value**		7.31; 6.9, (6.1, 8.1) N = 62663	6.75; 6.4, (5.8, 7.2) N = 28087	7.84; 7.4, (6.5, 8.9) N = 17469	7.27; 6.8, (6.1, 7.9) N = 9253	8.18; 7.9, (6.9, 9.2) N = 7854	< .0001	7.31; 6.9, (6.1, 8.1) N = 62715	7.30; 6.8, (6.1, 8.1) N = 56653	7.41; 7, (6.1, 8.3) N = 6062	< .0001
**A1c baseline**		7.42; 6.8, (6, 8.3) N = 62663	6.80; 6.3, (5.8, 7.3) N = 28087	8.02; 7.5, (6.5, 9.3) N = 17469	7.37; 6.8, (6.1, 8.1) N = 9253	8.39; 8, (6.9, 9.7) N = 7854	< .0001	7.42; 6.8, (6, 8.3) N = 62715	7.41; 6.8, (6, 8.3) N = 56653	7.52; 7, (6, 8.5) N = 6062	< .0001
**Quarterly type II diabetes related cost**		500.72; 126.2, (53.6, 291.4) N = 128791	263.39; 80.3, (28.2, 173.9) N = 59898	441.60; 145.8, (75.3, 315.3) N = 34471	768.16; 170.5, (85, 412.1) N = 19749	1228.09; 315.2, (158, 925.9) N = 14673	< .0001	484.22; 120.1, (46.8, 279.6) N = 133581	481.13; 119.9, (46.9, 276.2) N = 117680	507.07; 121.2, (46.2, 307) N = 15901	0.06
**Metro status**	1, metro	113530 (88%)	51728 (86%)	30659 (89%)	17813 (90%)	13330 (91%)	< .0001				
	2, non-metro	15261 (12%)	8170 (14%)	3812 (11%)	1936 (10%)	1343 (9%)					
**Complication type**	1, No							59898 (47%)	51728 (46%)	8170 (54%)	< .0001
	2, Short only							34471 (27%)	30659 (27%)	3812 (25%)	
	3, Long only							19749 (15%)	17813 (16%)	1936 (13%)	
	4, Short & Long							14673 (11%)	13330 (12%)	1343 (9%)	

* mean; median (interquartile range), sample size” are reported for continuous variables, and “frequency (column percentage)” are reported for categorical variables.

**Fig 2:**
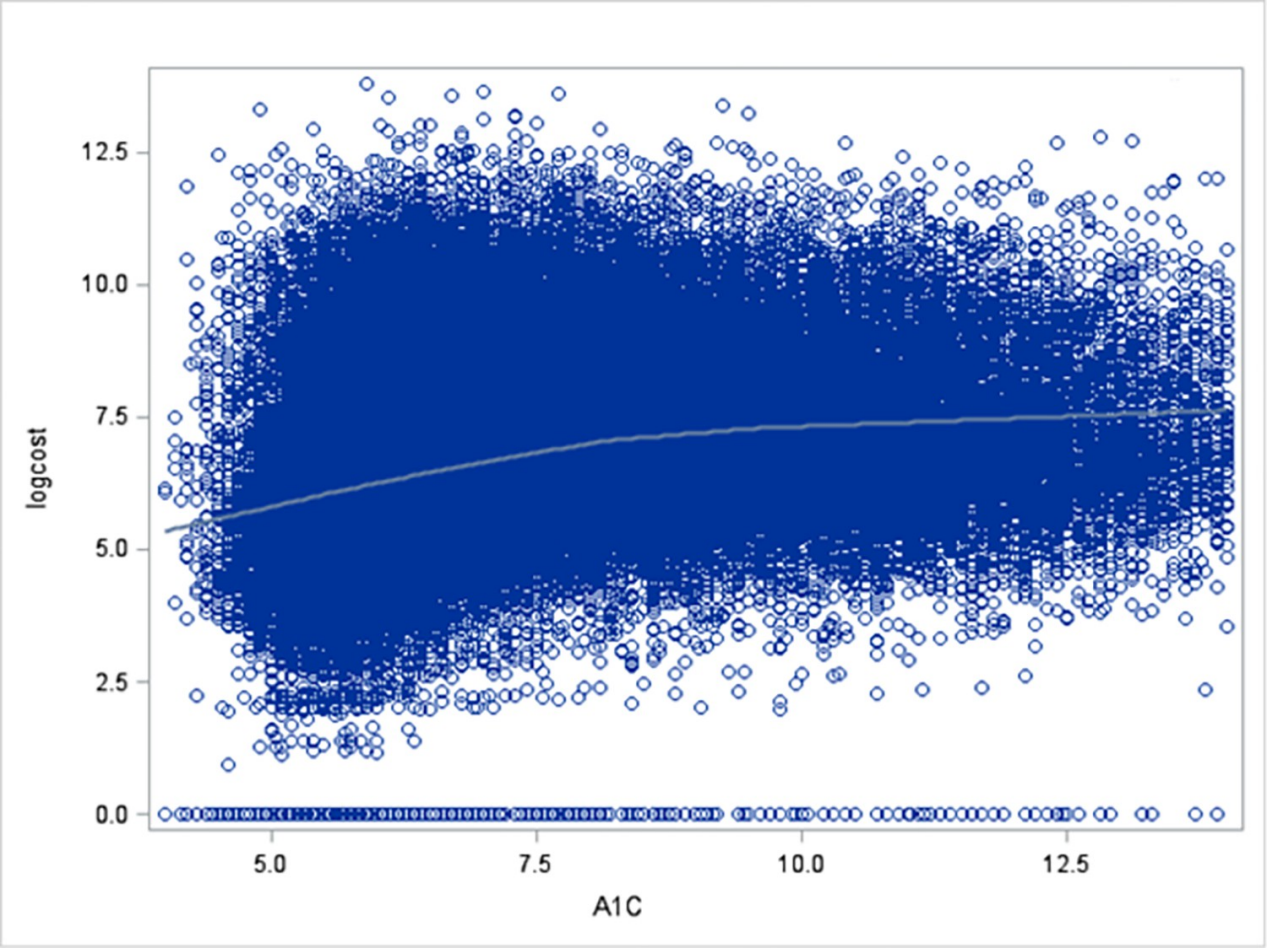
Diabetes-related costs for participants enrolled in 2018 and 2019 (log scale).

Fig 2 shows the bivariate relationship between A1c values and monthly diabetes-related cost, with cost on the log scale for easier visualization. We use a loess (locally estimated scatterplot smoothing estimate) to summarize the trend of diabetes-related cost at different A1c values. For patients with A1c values between 4.0% and 14.0%, the median diabetes-related cost was $955 per patient for 2 years. On average the costs increased with A1c values, and the speed of this increase (or the slope) was higher for A1C levels less than 8% than for A1c levels exceeded 8%. A higher slope in this figure indicates one unit increase of A1C corresponds to a higher fold change in the cost. The positive correlation between A1c and the diabetes-related cost was statistically significant.

Table 2 shows our fitted models for A1c values (Table 2A) and diabetes-related costs (Table 2B). In the multivariable longitudinal model examining A1c (Table 2A), the positive estimate of “Time in quarters” indicates that the A1c values increased over time. Men had higher A1c levels compared to women (estimate 0.048, 95% confidence interval or CI: 0.044 to 0.052). Relative to those with no diabetes-related complications, those with only short-term complications, only long-term complications, and both short- and long-term complications all had higher A1c values, where the estimates were 0.187, 0.102, and 0.277, respectively. Individuals with both short- and long-term complications generally had the highest A1c values. Those living in non-metro areas had higher A1c values than those living in metropolitan areas (estimate 0.036, 95% CI: 0.030 to 0.042).

**Table 2:** Factors associated with hemoglobin A1C values and diabetes-related costs. A. Factors associated with higher hemoglobin A1C values (on the log scale), B. Factors associated with higher diabetes-related cost (on the log scale).

**A.**					
**Factors**	**Level**	**Estimate**	**95% CI**		**P-value**
Time in Quarters		0.001	0.000	0.002	0.001
Age		-0.002	-0.002	-0.001	< .0001
Gender	male vs female	0.048	0.044	0.052	< .0001
Charlson Comorbidity Index (CCI)		-0.008	-0.010	-0.006	< .0001
Diabetes Complication	short-term vs. none	0.187	0.182	0.191	< .0001
	long-term vs. none	0.102	0.096	0.107	< .0001
	both vs. none	0.277	0.267	0.286	< .0001
Rurality	non-metro vs metro	0.036	0.030	0.042	< .0001
**B.**					
Time in Quarters		0.007	0.005	0.009	< .0001
Log A1c		1.323	1.217	1.430	< .0001
(Log A1c)^2^ second order		-0.283	-0.318	-0.249	< .0001
Age		-0.003	-0.004	-0.003	< .0001
Gender	male vs female	-0.033	-0.044	-0.023	< .0001
Charlson Comorbidity Index (CCI)		0.230	0.220	0.239	< .0001
Diabetes Complication	short-term vs. none	0.304	0.293	0.315	< .0001
	long-term vs. none	0.246	0.228	0.264	< .0001
	both vs. none	0.805	0.770	0.839	< .0001
Rurality	non-metro vs metro	-0.047	-0.064	-0.031	< .0001

In the multivariable longitudinal models for diabetes-related cost on the log scale (Table 2B), higher costs were associated with higher A1c values (estimate 1.323, 95% CI 1.217 to 1.430), younger age (estimate -0.003, 95% CI: -0.004 to -0.003), and higher CCI scores (estimate 0.230, 95% CI 0.220 to 0.239). Women had higher diabetes-related costs compared to men (male vs female estimate -0.033, 95% CI: -0.044 to -0.023). Relative to those with no diabetes-related complications, those with only short-term complications or long-term complications had approximately 35% and 28% higher diabetes-related costs, respectively, calculated as 35% = exp(0.304)-1 and 28% = exp(0.246)-1. Further, those with both short- and long-term complications had almost 123% higher diabetes-related costs compared to those with no complications (calculated as 123% = exp(0.805)-1). After adjusting A1c, age, sex, CCI, and complications in the model, metropolitan areas had higher diabetes-related costs than those living in non-metropolitan areas (non-metro vs metro: estimate -0.047, 95% CI: -0.064 to -0.031).

## Discussion

Study findings confirmed that demographic, geographic, and clinical risk factors and complications are associated with higher A1c values, especially among those residing in rural communities [23]. The findings suggest that even among the commercially insured individuals, common barriers such as limited access to healthcare and preventive health services [24] as well as foregoing care [24] can lead to diabetes-related complications, which are associated with higher diabetes-related costs. Further examination of contextual factors such as the role of social determinants of health at the aggregate level is warranted.

Study findings also reinforce the role of higher A1c values and complications as diabetes-related cost drivers [12]. As with other studies [25], we see a statistically significant positive relationship between A1c and the diabetes-related cost, although the slope of the increase becomes more gradual for A1c levels over .8. This relationship may be affected by substantial variation in costs, with some enrollees having minimal diabetes costs and others having nearly a million-dollar worth of diabetes related-costs. This variation calls for a further study of factors associated with the highest costs among the commercially insured patients. We also investigated the inpatient and outpatient diabetes-related costs and found majority of the patients had zero values in these two subcategories. Specifically, more than 95% and 75% had zero inpatient and outpatient diabetes-related costs, respectively. Future studies could investigate if the factors associated with higher diabetes related costs triggered inpatient, outpatient, or both costs.

We further examined factors supporting a linear relationship between A1c levels and health care costs [12, 24, 25]. Research suggests those without steady health insurance are more likely to delay or forego care [26] and such delayed or foregone care can be more costly, especially when a chronic condition such as diabetes requires continual clinical and home monitoring [27, 28]. The stability in cost may be associated with continuity of primary and specialty care that helps offset costly impatient and emergency room visits [12, 29]. Although Medicare has increased health care access dramatically, our research focus was on those under 65 who had commercial insurance, and hence access to a variety of preventive and monitoring services in different geographic settings. However, our study contributes to existing literature by suggesting a “shift” from A1c-focused diabetes management to diabetes-related complications management.

It is intriguing that despite living in non-metropolitan areas being associated with higher A1c values, higher diabetes-related costs were found in metropolitan areas. While this may appear counterintuitive, this finding may be explained by the higher cost of healthcare services in urban areas where provider contracts are likely to reimbursed at a higher rate. It is also possible that commercially insured individuals diagnosed with diabetes are more likely to reside in metro areas where they have fewer barriers to healthcare access. Texas still has one of the highest uninsured rates [30], making discussion of the potential coverage that may be gained through Medicaid expansion of interest, especially given the role insurance, in general, may play in access to care among those with diagnosed diabetes nationally [31]. It will also be important to track the impact of increased access to health insurance through the rapid rise of enrollment in government-sponsored health care exchanges.

The findings have clear implications for the importance of diabetes self-management to manage A1c levels and prevent short- and long-term complications. There needs to be greater coordination between health care systems that have limited time for in-depth diabetes education and community-based sectors which are delivering evidence-based diabetes self-management education and support shown to reduce A1c values and overall health care costs [24, 25]. We also note the importance of recognizing the social determinants of health that are specific to rural and underserved areas. Offering comprehensive diabetes self-management programs that include attention to community context in populations that are typically less reached can help reduce health disparities in A1c values which if left uncontrolled over long periods of time are likely to eventually result in more complications and higher costs.

### Limitations

The current analyses included one state, limiting generalizations to other states nationally. Further, the analysis sample includes commercially insured individuals from one insurance company, which further limits its external validity. However, it is important to note that we do have data from the largest private health insurer in Texas, which serves over 6 million members in all 254 TX counties. This plan also offers coverage across multiple lines of business, including employer group programs and Affordable Care Act (ACA) programs. We believe that makes our results applicable not only to TX commercial population, but also to comparable commercial populations in other states with large rural areas. We do concede that the implications of our results for publicly insured individuals (Medicare and Medicaid) are less precise as the sample excluded those 65 and older given medical costs were likely to be attributed elsewhere (e.g., Medicare). However, at the same time, a major strength was the use of statewide data that can inform key stakeholders and provide actionable information with which to use in planning efforts by multiple relevant sectors.

While administrative claims data provide information on a large population group, there are nevertheless inherent issues in this type of study relative to smaller clinically based studies which have their own limitations. For example, claims data do not document when diagnoses are initially made, potentially blurring the relationship between treatment and outcomes. Further, the plateauing of costs at higher A1C levels may reflect better management among those with commercial insurance coverage or, alternatively, that those in poorer control were using less care and that higher cost implications may appear at a later date.

Another potential limitation is the relatively short two-year time frame for this analysis. Although we are examining eight quarters of services, a longer time frame would be desirable to track changes in A1c values over time and their association with health care utilization, complications and costs. The shorter time frame was advantageous in minimizing geographic shifts from baseline that occurred in less than 1% of the population.

Additionally, the sample had more missing data than anticipated, specifically for the primary outcome variable A1c value. Missing lab values can be common in claim-based data even if a lab test was performed [32]. It was impossible to tell if missing data reflected that many enrollees with type 2 diabetes were not getting the recommended minimum of two professional A1c checks annually, or if these tests were done but simply not recorded in the claims database. The latter is a likely explanation since reporting A1c values was not required or reimbursed. A closer analysis of available A1c values indicated that 80% of the participants had two or less A1c values recorded in their administrative claims. Future research is needed to examine the overall health care utilization and costs associated with the frequency of A1c checks, controlling for other risk factors.

Further, information on race, ethnicity, education, and other relevant sociodemographic variables was not available in the data, a limiting factor, but not uncommon when using claims data. To counter this gap, we strongly recommend that administrative databases routinely collect data that can be used to assess social determinants of health. This is critical given the key influence of social and structural factors on inequities [33] and their strong relationship to morbidity and mortality outcomes [34]. Further studies should concentrate on adding Medicare, Medicaid, and uninsured populations to see how their outcomes compare to commercially insured populations. In addition, extending the study period to the post-COVID-19 era would help to analyze the impacts of the pandemic on healthcare costs given an increase in unmanaged diabetes as well as complications such as diabetic ketoacidosis [35].

## Conclusion

This study evaluates factors associated with A1c and diabetes-related costs. The study is unique in its focus on a subset of commercially insured adult individuals with type 2 diabetes residing in a populous and growing state with substantial socio-demographic diversity and large concentrations of population in both urban and rural areas. Focusing on enrollees under 65 with a mean age of approximately 50 years of age, our analysis of the health outcomes and health utilization of the adult population provides valuable insights and guides preventive care for our rapidly aging population in Texas and throughout the U.S.
